# Case Report: Efficacy of Rituximab in a Patient With Familial Mediterranean Fever and Multiple Sclerosis

**DOI:** 10.3389/fneur.2020.591395

**Published:** 2021-01-06

**Authors:** Mattia Pozzato, Emanuele Micaglio, Chiara Starvaggi Cucuzza, Alessandro Cagol, Daniela Galimberti, Daniela Calandrella, Claudia Cinnante, Carlo Pappone, Monica Zanussi, Giovanni Meola, Elio Scarpini, Nereo Bresolin, Filippo Martinelli Boneschi

**Affiliations:** ^1^Foundation Istituto di Ricovero e Cura a Carattere Scientifico (IRCCS) Policlinico San Donato, Ca' Granda Ospedale Maggiore Policlinico, Neurology Unit & MS Centre, Milan, Italy; ^2^Neuroscience Section, Department of Pathophysiology and Transplantation (DEPT), Dino Ferrari Centre, University of Milan, Milan, Italy; ^3^Arrhythmology and Electrophysiology Department, Istituto di Ricovero e Cura a Carattere Scientifico (IRCCS) Policlinico San Donato, Milan, Italy; ^4^Department of Neurology, Istituto di Ricovero e Cura a Carattere Scientifico (IRCCS) Policlinico San Donato, San Donato Milanese, Italy; ^5^Department of Biomedical, Surgical and Dental Sciences, Dino Ferrari Centre, University of Milan, Milan, Italy; ^6^Department of Neurology, Istituto di Ricovero e Cura a Carattere Scientifico (IRCCS) Policlinico San Donato, Humanitas Research Hospital and University, Milan, Italy; ^7^Neuroradiology Unit, Foundation Istituto di Ricovero e Cura a Carattere Scientifico (IRCCS) Policlinico San Donato, Ca' Granda Ospedale Maggiore Policlinico, Milan, Italy; ^8^Clinical Genomics-Molecular Genetics Service, Istituto di Ricovero e Cura a Carattere Scientifico (IRCCS) Policlinico San Donato, San Raffaele Hospital, Milan, Italy; ^9^Department of Biomedical Sciences for Health, University of Milan, Milan, Italy; ^10^Department of Neurorehabilitation Sciences, Casa di Cura Privata del Policlinico, Milan, Italy

**Keywords:** multiple sclerosis, rituximab, hepatotoxicity, case report, familal mediterranean fever

## Abstract

Familial Mediterranean Fever (FMF) is a genetic autoinflammatory disease characterized by recurrent episodes of fever and serositis caused by mutations in the MEFV gene, while Multiple Sclerosis (MS) is an inflammatory demyelinating disease of the CNS with genetic and environmental etiology. The two diseases rarely occur in association with relevant implications for clinical management and drug choice. In this paper, we present the case of a 53-year-old male with an autosomal dominant FMF since childhood who presented acute paresthesia at the right part of the body. He performed a brain and spinal cord MRI, which showed multiple brain lesions and a gd-enhancing lesion in the cervical spinal cord, and then received a diagnosis of MS. He then started Interferonβ-1a which was effective but not tolerated and caused hepatotoxicity, and then shifted to Rituximab with 3-month clinical and neuroradiological efficacy.

## Background

Familial Mediterranean Fever (FMF) is an inherited disease caused by mutations in *MEFV* (Mediterranean fever) gene, which encodes the pyrin protein, an important modulator of innate immunity. *MEFV* gene is localized on chromosome 16p13.3 and consists of 10 exons. Five founder mutations (M680I, M694V, M694I, V726A, E148Q) account for over 85% of Mediterranean-origin based FMF cases, with M694V mutation being the most common (1), associated with worse prognosis and higher risk of comorbidity.

FMF is commonly reported in the Mediterranean region, with a prevalence of 1:150–1:10.000 (2). The reason is likely the so called “founder effect,” which is the migration of a small group of people, in this case of Jewish origin, from a larger population to go settling in another environment.

The clinical picture of FMF shows an extreme grade of variability (3), from asymptomatic carrier of pathogenic mutations (4) to patients with various autoinflammatory symptoms (fever, skin erythema, multi-articular arthritis, serositis) or renal amyloidosis even without fever. Moreover, FMF can be complicated by the co-occurrence of other autoimmune diseases, such as rheumatic inflammatory diseases and Multiple Sclerosis (MS) (5).

MS is an inflammatory demyelinating disease of the Central Nervous System (CNS) with complex etiology due to a combination of genetic susceptibility driven by > 200 genetic loci (6) and environmental triggers (hypovitaminosis-D, smoking and obesity), resulting in a self-sustaining autoimmune disorder with immune cell infiltration and release of inflammatory cytokines in CNS (7). The adverse drug reaction rate is considerably high in MS, due to the complexity of clinical management.

Previous works already described the co-occurrence of FMF and MS in same patients but little is known on the best therapy (5, 8, 9). The most common therapeutical approaches used are Interferonβ-1a, because of its ability to down-regulate pro-inflammatory cytokines, and Natalizumab, due to its ability to reduce the entry of autoimmune cells into CNS.

## Case Report

**Figure 1:**
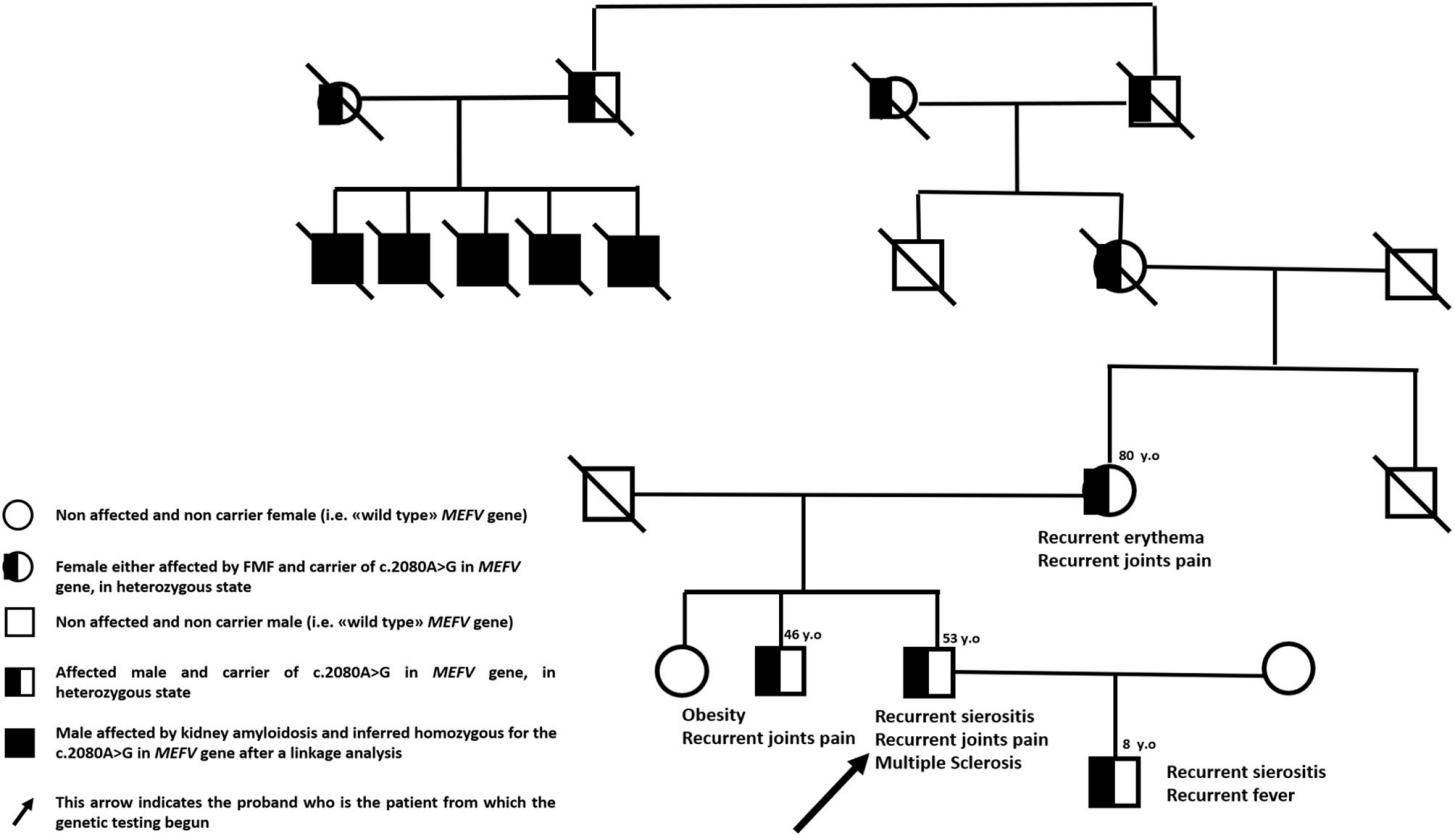
Family pedigree.

We describe the case of a 53-year-old man with Sephardic Jewish descent from either side of family-tree and a personal history of FMF. Five first-grade cousins of his maternal grandmother presented kidney amyloidosis at young age, his mother suffers from recurrent skin rashes and his brother is affected by multiarticular arthritis since young age. Moreover, proband's only 8-year-old male child is affected by a classical FMF phenotype with recurrent serositis and self-limiting fever attacks (Figure 1). In proband's history, the disease started at the age of four with acute episodes of fever, asthenia and abdominal pain but he received the diagnosis of FMF at 40 years of age after the suspicion emerged in his son. Since the diagnosis, patient is taking colchicine 1.5 mg daily with complete remission of inflammatory symptoms.

**Figure 2:**
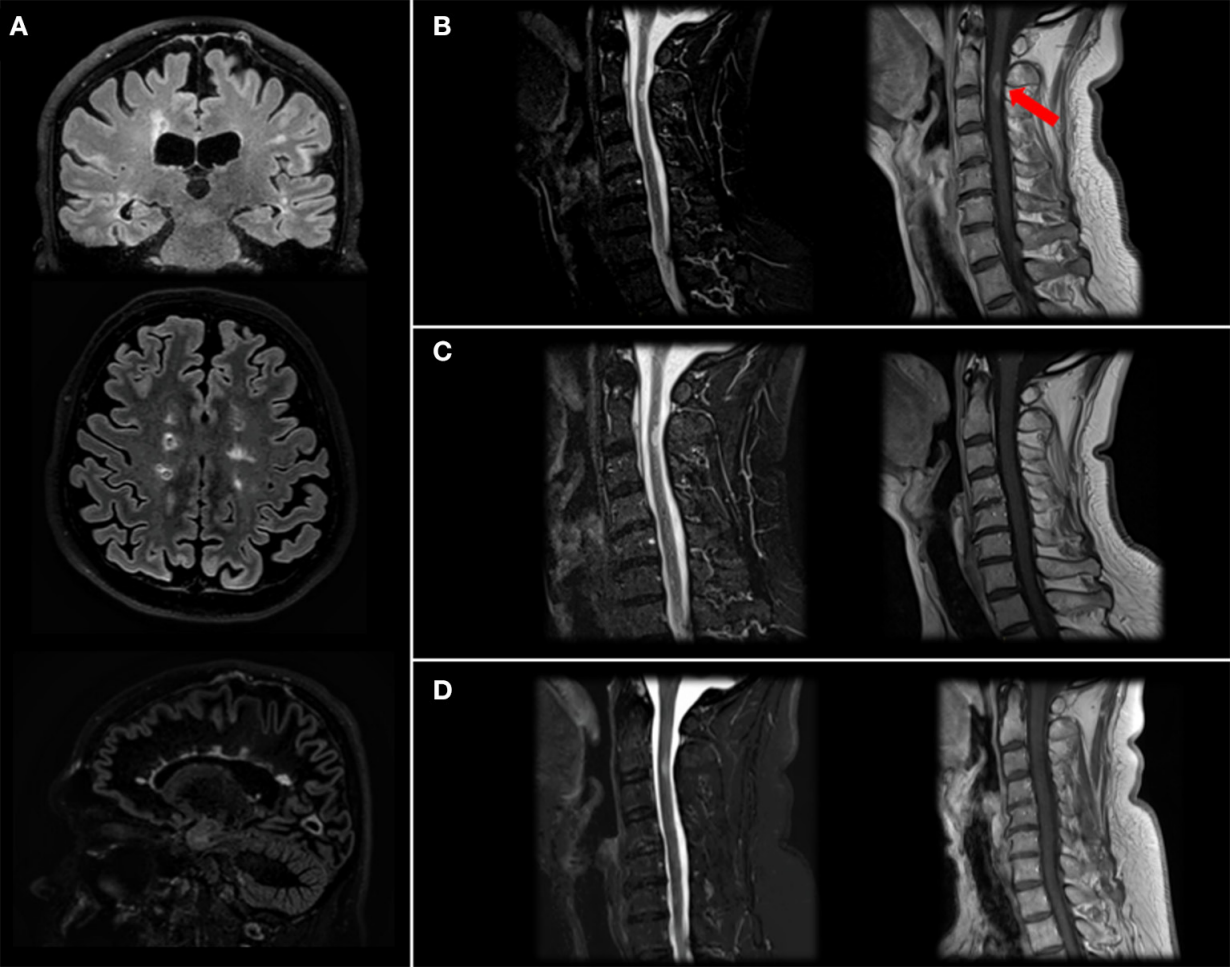
Brain and spinal cord MRI. Brain MRI images (Fluid Attenutated Inversion Recovery coronal and axial projections, Double Inversion Recovery sagittal projection) showing typical MS lesions involving bi-hemispheric white matter **(A)**. Cervical MRI image (Short Time Inversion Inversion Recovery and T1-gd+ sagittal projection) at diagnosis **(B)** showing a C2-C3 level lesion with gd-enhancement (arrow), resolution after steroid treatment **(C)** and stability at 4-month follow-up after two cycles of Rituximab **(D)**.

Patient has come to neurologic consultation in 2/2019 because of an acute episode of itch and tingling paresthesia involving the right part of the body and urinary urgency. He performed a brain and spinal cord MRI, which showed multiple supra- and infratentorial lesions (Figure 2A) and a gadolinium-enhancing lesion in the cervical spinal cord at right lateral C2-C3 level responsible for symptoms (Figure 2B).

CSF analysis revealed specific oligoclonal band positivity, and we made a diagnosis of relapsing-remitting MS according to McDonald revised diagnostic criteria (10). Serological screening for systemic autoimmunity was negative, and liver and renal functions were normal without any biochemical signs of liver pathology.

An additional genetic screening has been performed for confirming FMF genetic diagnosis after the patient signed an informed consent (protocol number IRCCS Ca' Granda Ospedale Maggiore Policlinico: M.33.F, date 13/1/2020). By using NGS technology, we sequenced the following genes involved in autoinflammatory diseases: *ELANE, IL10RA, IL10RB, IL1RN, IL36RN, KHDC3L, LPIN2, MEFV, MVK, NCSTN, NLRP12, NLRP3, NLRP7, NOD2, PDCD1, PLCG2, PSENEN, PSMB8, PSTPIP1, SH3BP2, TNFRSF1A*. Median coverage was 89X with only 4 genic regions with coverage <10X in the coding regions of *NLRP12* gene (chr 19, region 54312841–54313290) and *SH3BP2* gene (chr 4, regions 2819941–2820117, 2831830–28311289 and 2831220–2831289)]. Genetic testing confirmed the c.2080A>G (p. Met694Val) mutation in a single copy of *MEFV* gene with a confirmed maternal inheritance. The same mutation has been detected also in proband's son. The other five relatives (living outside of Italy) underwent a linkage analysis in early nineties showing a little homozygosity region on the short arm of chromosome 16 (16p13.3). These findings contributed later to the definition of *MEFV* gene position in human genome (11).

Neurological examination performed at time of MS diagnosis showed abnormalities in extrinsic ocular motility (deficit of adduction of the left eye and inconstant horizontal diplopia on right gaze), bilateral lower limbs hypopallestesia and mild right upper and lower limb weakness, leading to a disability of 3.5 on EDSS scale.

We treated the patient with a high-dose of steroids with benefit on motor impairment and diplopia, while sensory symptoms were still present (EDSS: 3.5). One month-follow up MRI was stable in terms of lesion burden, and the cervical lesion no longer showed gd-enhancement (Figure 2C).

Because of patient's positivity to anti-JCV antibody at high titer (> 1.5) and the associated risk of multifocal progressive leukoencephalopathy, he started Interferon β-1a (IFN) treatment in 4/2019, also considering the concomitant colchicine assumption. Following MRI and neurological examinations were stable.

Unfortunately, since the beginning the patient presented severe flu-like syndrome episodes and an elevation in liver enzymes (AST: 60 U/L; ALT: 111 U/L), and the drug was interrupted in 12/2019 after 9 months.

Therefore, we decided, also considering the high brain and spinal cord lesional load, to switch the patient to Rituximab, performing two cycles of 500 mg. Two weeks apart, that he performed in 2/2020, and maintenance doses of 500 mg. total every 6 months. As expected, at second cycle we observed a depletion of peripheral blood CD19+ B cells (20.2% to 0%) and a mild reduction of absolute levels of CD4+ and CD8+ cells. Neurofilaments light chain (NfL) levels, measured before Rituximab start using a highly sensitive electrochemiluminescence based immunoassay (Meso Scale Discovery) were 31 pg/ml, in line with previous studies MS (12). No additional lesions were detected at last brain and cervical spinal cord MRI performed in 6/2020 (Figure 2D). Last blood examinations in 9/2020 showed that liver tests reverted within normal limits and CD19+ were 0; patient has a stable EDSS, no new relapses and an improved perceived health status and he received a new cycle with 500 mg. of rituximab with no adverse events.

## Discussion

FMF and MS are inflammatory conditions involving different types of immunity, FMF the innate and MS the adaptive immune system.

Mutant alleles of the *MEFV* gene are more common among Jewish Sephardic population, with an estimated frequency of about 1/8,000 individuals compared to 1/24,000 in Ashkenazi, while MS is less common in Sephardic, supporting the hypothesis that the MS-risk in this patient of Sephardic origin was increased by the presence of *MEFV* mutation, despite a lower presence of MS-risk alleles (13, 14) and the absence of the HLA-DRB1^*^1501 which is the main genetic risk factor for MS (14). Our case was the only subject in the family with MS, and given the complex etiology of the disease also driven by environmental factors, like low vitamin D levels, we supported a primary prevention strategy for his son who carries the same mutation.

The MS-FMF association has already been analyzed in large cohorts of FMF subjects, establishing that the prevalence of MS in FMF patients is more than twice higher than in the general population (*p* < 0.005) and that M694V homozygosity in *MEFV* gene is associated with a higher risk of MS (*p* < 0.005), a more frequent progressive MS course and a quicker disability progression (9).

The association of FMF with MS and other inflammatory rheumatic conditions can be due to an increased inflammatory milieu, since pyrin is implicated in the maturation and secretion of IL-1β, a major mediator of fever and systemic inflammation. Indeed, mutations in certain domains of the protein are thought to cause uncontrolled IL-1β release, resulting in systemic inflammation. Moreover, IL-1β has been shown to be involved in the process of demyelination in EAE MS mouse model (15).

Colchicine is the main treatment approved for FMF, due to its proven efficacy in reducing frequency of attacks. In colchicine non-responder patients, biologic agents which inhibits IL-1 (16), IL-6 (17), and TNF-alpha (18) are used.

There are no data on IL-1 receptor antagonists use in MS patients, while the IL-6 receptor antagonist Tocilizumab is currently used in NMOSD as a second-line (17) but not in MS, and TNF-alpha inhibitors has been shown to be detrimental to MS (18), rendering their use not appropriate in cases with FMF and MS.

Few studies on FMF-MS cases showed a beneficial use of IFN and Glatiramer Acetate (GA). In one paper on a Israeli registry of 12.000 FMF patients, 9 MS patients have been identified and 5 treated with IFN and 4 with GA with only one shift from IFN to GA to Azathioprine (9). There is then a clinical report on an Italian case of FMF-MS who was shifted from IFN to Natalizumab, for whom no data on follow-up are available (19).

Interferon β-1a is a well-tolerated drug, but adverse events like injection-site reactions, flu-like syndrome and liver enzymes elevation are relatively common and can lead to discontinuation of therapy (20). Natalizumab is an alternative second-line option, but in this case it was not chosen due to the high anti-JCV titer of the patient. Therefore, alternative options were azathioprine, for which however no clinical trials are available in MS, or more effective options like anti-CD20 drugs.

Hamano et al. (21) evaluated Rituximab, a human-mouse chimeric monoclonal antibody directed against the CD20 antigen on mature B-cells, in a patient affected by a nephrotic syndrome due to membranoproliferative glomerulonephritis with an history of periodic fever syndrome of unknown origin, unresponsive to steroid therapy and immunosuppressants, with an improvement of renal function, a remission of the nephrotic syndrome and no adverse events. Although no clinical studies have been conducted, Rituximab use in FMF could lower the production of cytokines overexpressed in this pathology by depleting the B lymphocyte count. Moreover, the efficacy of the drug in reducing relapse frequency and MRI activity has been demonstrated in different studies in MS (22). Our case further supported the efficacy of the drug.

## Conclusion

In this paper, we used an anti-CD20 drug in a genetically confirmed FMF patient with MS. At 4-month follow-up, we can say that the drug is safe, well-tolerated and effective.

Given the recent change in therapeutic algorithm in MS patients, we can support this option in FMF-MS patients who are non-responders or develop adverse reactions to first-line drugs.

We finally support the need to create a registry on patients affected with more than one inflammatory disorders to help selecting drugs targeting the immune system.

## Ethics Statement

The studies involving human participants were reviewed and approved by IRCCS Ca' Granda Ospedale Maggiore Policlinico. The patients/participants provided their written informed consent to participate in this study. Written informed consent was obtained from the individual(s) for the publication of any potentially identifiable images or data included in this article.

## Author Contributions

MP, EM, and FM conceptualization, writing the full text, and fundraising. CP helped the authors to reach stronger conclusions. CS, AC, DG, DC, CC, MZ, GM, ES, and NB read, approved, and subscribed final version. All the authors read and approved the final manuscript, and all included figures.

## Conflict of Interest

The authors declare that the research was conducted in the absence of any commercial or financial relationships that could be construed as a potential conflict of interest.
